# Dose- and Time-Dependent Effects of Oleate on Mitochondrial Fusion/Fission Proteins and Cell Viability in HepG2 Cells: Comparison with Palmitate Effects

**DOI:** 10.3390/ijms22189812

**Published:** 2021-09-10

**Authors:** Isy F. de Sousa, Vincenzo Migliaccio, Marilena Lepretti, Gaetana Paolella, Ilaria Di Gregorio, Ivana Caputo, Eliane Beraldi Ribeiro, Lillà Lionetti

**Affiliations:** 1 Escola Paulista de Medicina, Departamento de Fisiologia, Universidade Federal de São Paulo, São Paulo 04023-062, SP, Brazil; isyfs18@gmail.com (I.F.d.S.); eliane.beraldi@gmail.com (E.B.R.); 2 Dipartimento di Chimica e Biologia “A. Zambelli”, Università degli Studi di Salerno, 84084 Fisciano, SA, Italy; vmigliaccio@unisa.it (V.M.); mleprettil@gmail.com (M.L.); gpaolella@unisa.it (G.P.); idigregorio@unisa.it (I.D.G.); icaputo@unisa.it (I.C.); 3 European Laboratory for the Investigation of Food-Induced Diseases (ELFID), University of Salerno, 84084 Fisciano, SA, Italy

**Keywords:** mitochondrial dynamics, monounsaturated fatty acids, saturated fatty acids, hepatocytes, cellular adaptation, lipotoxicity, MFN2, DRP1

## Abstract

Mitochondrial impairments in dynamic behavior (fusion/fission balance) associated with mitochondrial dysfunction play a key role in cell lipotoxicity and lipid-induced metabolic diseases. The present work aimed to evaluate dose- and time-dependent effects of the monounsaturated fatty acid oleate on mitochondrial fusion/fission proteins in comparison with the saturated fatty acid palmitate in hepatic cells. To this end, HepG-2 cells were treated with 0, 10 μM, 50 μM, 100 μM, 250 μM or 500 μM of either oleate or palmitate for 8 or 24 h. Cell viability and lipid accumulation were evaluated to assess lipotoxicity. Mitochondrial markers of fusion (mitofusin 2, MFN2) and fission (dynamin-related protein 1, DRP1) processes were evaluated by Western blot analysis. After 8 h, the highest dose of oleate induced a decrease in DRP1 content without changes in MFN2 content in association with cell viability maintenance, whereas palmitate induced a decrease in cell viability associated with a decrease mainly in MFN2 content. After 24 h, oleate induced MFN2 increase, whereas palmitate induced DRP1 increase associated with a higher decrease in cell viability with high doses compared to oleate. This finding could be useful to understand the role of mitochondria in the protective effects of oleate as a bioactive compound.

## 1. Introduction

Impairments in mitochondrial bioenergetics and dynamics behavior play a key role in hepatic pathology associated with high fat feeding and obesity, such as insulin resistance and non-alcoholic fatty liver disease (NAFLD) [1,2,3]. Hepatic injury occurs when hepatocyte capacity to cope with an increased level of circulating fatty acids is exceeded [4,5,6,7], and substrate oversupply triggers cellular stress with impairment in mitochondrial and endoplasmic reticulum (ER) homeostasis [6,7,8,9,10,11,12].

Mitochondrial function is closely related to morphology and dynamic behavior, in terms of mitochondrial fusion and fission processes. The fusion process is regulated by mitofusin 2 (MFN2), which coordinates the fusion of the outer mitochondrial membrane, and by optic atrophy 1 (OPA1), which coordinates the fusion of the inner mitochondrial membrane. The fission process is regulated by dynamin-related protein 1 (DRP1) and fission protein 1 (FIS1) [13]. The shift of mitochondrial fusion/fission balance toward fission processes is associated with mitochondrial dysfunction, oxidative stress and apoptosis, as well as with hepatic steatosis and insulin resistance onset. On the other hand, a shift toward fusion process is associated with the prevention of these metabolic alterations [11,12,13]. The scarcity of nutrients can promote either mitochondrial fusion or fission, whereas an overload of glucose promotes fission accompanied by elevated reactive oxygen species production [14]. Hepatic ablation of MFN2 elicited glucose intolerance and impairment in hepatic insulin signaling [15]. In diet-induced obesity, impaired mitochondrial function and increased fission processes were found in liver and skeletal muscle [11,16,17]. Diverse dietary fat sources affect mitochondrial dynamics and bioenergetics differently. Saturated fatty acids induced a shift toward fission processes in liver and skeletal muscle from rats fed a high lard diet compared to control rats [16,17]. In contrast to saturated fatty acids, omega 3 polyunsaturated fatty acids induced fusion processes and improved mitochondrial function both in in vivo and in vitro experimental models (reviewed in [11]).

Mitochondrial dysfunction and disruption in mitochondrial dynamics are associated with endoplasmic reticulum (ER) stress. Under conditions of lipid oversupply, ER homeostasis is impaired, and unfolded protein response (UPR) is activated [6,7,8,9]. UPR aims to restore normal cell function by interrupting protein translation, degrading misfolded proteins and/or activating pathways to increase the production of chaperones involved in protein folding, such as glucose regulatory protein 78 (GRP78). However, if none of the above-mentioned mechanisms can effectively counteract the failure of protein-folding process, apoptosis pathways will be recruited [8,18]. Toxic lipids can directly activate UPR sensors, independently by the accumulation of misfolded proteins [19,20]. A long-term activation of UPR could lead to metabolic alterations associated with high fat feeding, such as NAFLD and diabetes mellitus 2 [20].

Diverse types of dietary fatty acids differently contribute to hepatic lipotoxicity in the etiopathogenesis of NAFLD and insulin resistance [11]. Palmitate, the most abundant saturated fatty acid consumed in a typical Western-pattern diet, has been pointed out as the main fatty acid responsible for hepatic lipotoxicity and insulin resistance [21,22,23,24,25,26]. Saturated fatty acids induce impairments in mitochondrial function and dynamic behavior as well as in ER homeostasis [6,21,27]. On the other hand, unsaturated fatty acids have been shown to be less toxic. In particular, it has been suggested that omega 3 polyunsaturated fatty acids have a protective effect on NAFLD and diabetes, by modulating mitochondrial bioenergetics, dynamic behavior, and ER stress [28,29,30,31]. Moreover, oleic acid, the predominant component in olive oil (70–80%) which in turn is the main fat source in the Mediterranean diet, seems to have protective effects on insulin resistance and NAFLD onset [26,32,33,34,35]. It has been shown that oleic acid is able to prevent oxidative and ER stress caused by palmitic acid [26]. Oleic acid supplementation in cells treated with palmitic acid recovered mitochondrial dysfunction caused by the saturated fatty acid [35]. In HepG2 cells, saturated fatty acids reduced ATP production in a dose-dependent manner, by impairing OXPHOS complexes activity. Moreover, cells treated with saturated fatty acids showed an elevated production of oxidative stress markers. On the other hand, cells incubated with oleic acid did not present any change in ATP production or thiobarbituric-acid-reacting substances (TBARS) levels [33]. Of note, the coincubation of oleic acid with palmitic acid was also able to revert the impairments caused by saturated fatty acids. Therefore, the literature data support the hypothesis that oleic acid, unlike saturated fatty acids, has protective effects against lipotoxicity, mitochondrial dysfunction and ER stress, but as far as we know, little is known about the involvement of mitochondrial dynamics mechanisms. The main aim of the present study was to investigate the dose- and time-dependent effect of oleate on contents of MFN2, as a mitochondrial marker of fusion processes, and DRP1, as a marker of fission processes, compared to palmitate in HepG2 cells. The analyses of dose- and time-dependent fatty acids impact on mitochondrial dynamic proteins are associated with the analyses of dose- and time dependent effects on cell viability and lipid accumulation. Apoptosis and early ER stress markers (namely GRP78) have also been assessed. Analyzing fatty acids dose- and time-dependent effects on these parameters could be useful: (1) to understand the role of mitochondrial dynamic processes in cellular physiological adaptation to increasing doses or time of exposure to stressor agents, such as dietary fatty acids; (2) to clarify the mechanisms by which cells shift from a physiological adaptation to a condition of marked stress by inducing cell death/apoptosis in response to stressor agents. This kind of study could also shed more light on cellular pathways activated by nutrients, such as oleic acid, with protective effects on metabolic diseases.

## 2. Results

### 2.1. Comparison of Dose- and Time-Dependent Effects of Oleate and Palmitate on Cell Viability and Lipid Accumulation

To compare dose- and time-dependent cytotoxic effects of oleate and palmitate in hepatocytes, HepG2 cells were incubated with different concentrations of oleate or palmitate (0 μM, 10 μM, 50 μM, 100 μM, 250 μM and 500 μM) for 8 or 24 h, and then analyzed by 3-(4.5-dimethylthiazol-2-yl)-2.5-diphenyltetrazolium bromide (MTT) assay.

**Figure 1:**
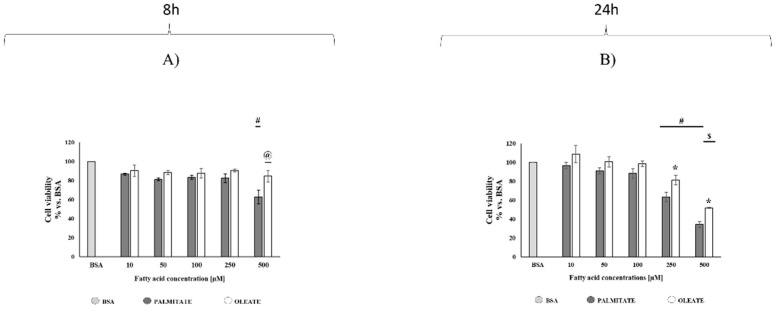
Dose- and time-dependent effects of oleate or palmitate on HepG2 cells viability. Cells were treated with different doses of oleate or palmitate (0 μM, 10 μM, 50 μM, 100 μM, 250 μM and 500 μM) for 8 (**A**) or 24 (**B**) h. All data are presented as mean ± standard error of the mean (SEM) for 3 biological replicates. (**A**) After 8 h, two-way ANOVA analyses showed that the fatty acids effect was very significant (*p* = 0.0032), concentrations effect was extremely significant (*p* < 0.0001), and interaction was not significant. # *p* < 0.05 palmitate vs. bovine serum albumin (BSA) and vs. lower palmitate concentrations. @ *p* < 0.05 oleate vs. palmitate according to a Tukey post hoc multiple comparison test. One-way ANOVA analyses confirmed dose-dependent effect for palmitate, but not for oleate. (**B**) After 24 h, two-way ANOVA analyses showed that fatty acids effect was very significant (*p* = 0.0002), concentrations effect was extremely significant (*p* < 0.0001) and interaction was not significant. # *p* < 0.05 palmitate vs. BSA and vs. lower palmitate concentrations, $ *p* < 0.05 oleate vs. BSA and vs. lower oleate concentrations according to a Tukey post hoc multiple comparison test. One-way ANOVA analysis confirmed the dose-dependent effect on cell viability for both palmitate and oleate. Student’s *t*-test: * *p* < 0.05 oleate vs. palmitate.

After 8 h, oleate did not induce any changes in cell viability, whereas the highest dose of palmitate elicited a significant cytotoxic effect by reducing cell viability to a value of 63% vs. bovine serum albumin (BSA) control cells (100%) (Figure 1A). Therefore, 500 µM oleate-treated cells showed a significantly high viability (+33%) compared to palmitate-treated cells. The palmitate cytotoxic effect was even more marked after 24 h, where cell viability was reduced to the value of 63% and 34% vs. BSA control cells (100%) with the doses of 250 and 500 μM, respectively (Figure 1B). Oleate-treated cells also showed dose-dependent decreases in cell viability after 24 h, reaching a value of 81% and 52% vs. BSA cells with the doses of 250 and 500 μM, respectively (Figure 1B). It should be noted that 250 and 500 μM oleate-treated cells showed a significantly higher cell viability (+28% and +53%, respectively) compared to 250 and 500 μM palmitate-treated cells (Figure 1B).

Spectrophotometric and microscopy analyses confirmed that oleate and palmitate induced a progressive lipid accumulation in a dose- and time-dependent manner.

**Figure 2:**
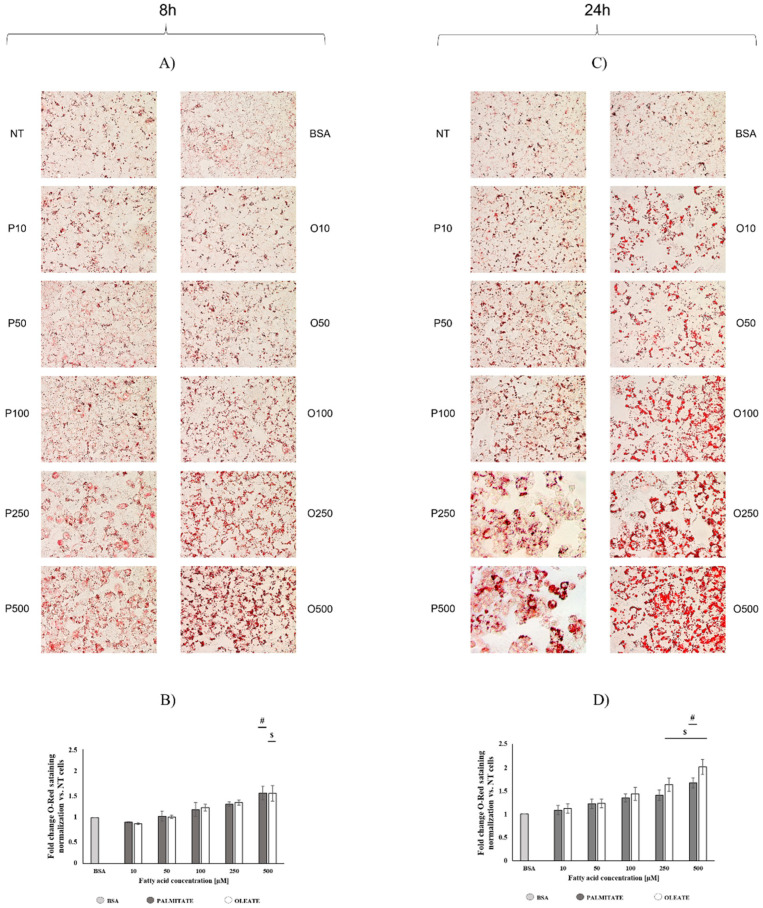
Dose- and time-dependent effect of oleate or palmitate on cell lipid accumulation. Cells were treated with different doses of oleate or palmitate (0 μM, 10 μM, 50 μM, 100 μM, 250 μM and 500 μM) for 8 or 24 h and stained by using oil red analyses. (**A**,**C**) Lipid deposits in cells were detected by using a microscope fitted with a camera. Original images were obtained using 40× magnification. (**B**,**D**) Quantitative analyses of lipid deposition was presented as mean ± SEM for 3 or more biological replicates. (**B**) After 8 h, two-way ANOVA test showed that concentrations effect was extremely significant (*p* < 0.0001); fatty acids effect and interaction were not significant. # *p* < 0.05 palmitate vs. BSA and vs. lower palmitate concentrations, $ *p* < 0.05 oleate vs. BSA and vs. lower oleate concentrations according to a Tukey post hoc multiple comparison test. One-way ANOVA analyses confirmed dose-dependent effects for both oleate and palmitate. (**D**) After 24 h, two-way ANOVA test showed that concentrations effect was extremely significant (*p* < 0.0001), fatty acids effect and interaction were not significant. # *p* < 0.05 palmitate vs. BSA and vs. lower palmitate concentrations, $ *p* < 0.05 oleate vs. BSA and vs. lower oleate concentrations according to a Tukey post hoc multiple comparison test. One-way ANOVA analyses confirmed dose-dependent effect for both oleate and palmitate.

After 8 h of exposure, oleate induced a dose-dependent increase in lipid accumulation, reaching a significant 50% increase in 500 μM treated cells vs. BSA (Figure 2B). A similar trend was observed in palmitate-treated cells. After 24 h, a further dose-dependent increase in lipid accumulation was detected (Figure 2D). Oleate induced significant increases (+66% and +100%) in cellular lipid deposits in 250 μM and 500 μM treated vs. BSA cells, respectively, whereas palmitate induced a significant increase (+66%) in 500 μM treated vs. BSA cells.

Cell lipid accumulation was associated with morphological changes mainly in palmitate-treated cells. After 24 h, oleate-treated cells did not show any marked morphological variations associated with lipid accumulation, whereas palmitate-treated cells showed a change in their morphology by adopting a round shape mainly with high doses (250 and 500 µM) (Figure 2C).

### 2.2. Comparison of Dose- and Time-Dependent Effects of Oleate and Palmitate on Mitochondrial Fusion and Fission Protein Markers

To compare dose- and time-dependent effects of oleate and palmitate on mitochondrial dynamic behavior, key proteins involved in mitochondrial dynamic machinery, namely MFN2 as a marker of fusion process and DRP1 as a marker of fission process, were analysed by Western blot.

**Figure 3:**
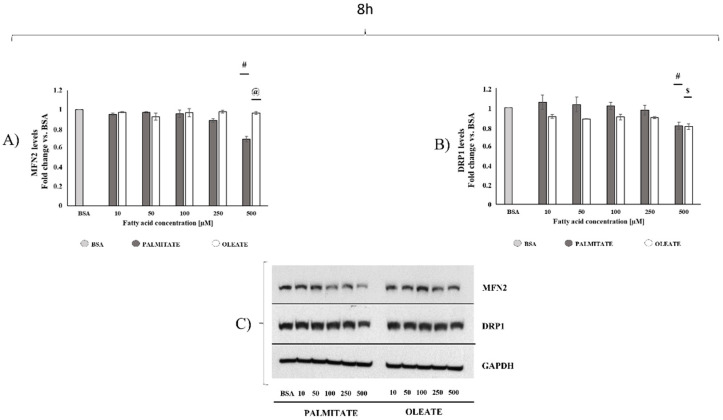
Mitochondrial dynamics markers after 8 h of exposure. Cells were treated with different doses of oleate or palmitate (0 μM, 10 μM, 50 μM, 100 μM, 250 μM and 500 μM) for 8 h. All data are presented as mean ± SEM for 4 biological replicates for mitofusin (MFN2) (**A**) and 3 biological replicates for dynamin-related protein 1 (DRP1) (**B**). (**A**) For MFN2, two-way ANOVA analysis showed extremely significant concentrations effect (*p* < 0.0001), fatty acids effect (*p* = 0.0001), and interaction (*p* < 0.0001). # *p* < 0.05 palmitate vs. BSA and vs. lower palmitate concentrations, @ *p* < 0.05 oleate vs. palmitate according to a Tukey post hoc multiple comparison test. One-way ANOVA analyses confirmed a dose-dependent effect only for palmitate. (**B**) As for DRP1, two-way ANOVA analysis showed an extremely significant concentration effect (*p* < 0.0001) and fatty acids effect (*p* = 0.0006), with no significant interaction. # *p* < 0.05 palmitate vs. BSA and vs. lower palmitate concentrations, $ *p* < 0.05 oleate vs. BSA according to a Tukey post hoc multiple comparison test. One-way ANOVA analyses confirmed dose-dependent effects for both oleate and palmitate. (**C**) Representative images of MFN2, DRP1 and loading control for protein normalization glyceraldehyde 3-phosphate dehydrogenase (GAPDH) were reported.

After 8 h, oleate did not elicit any dose-dependent changes in MFN2 content, whereas palmitate induced a significant MFN2 content decrease (−30%) in 500 μM treated cells vs. BSA. Therefore, MFN2 content was significantly higher (+39%) in 500 μM oleate-treated cells compared to palmitate-treated cells (Figure 3A). Regarding fission processes, oleate elicited a slight decrease (about 10%) starting by the lowest dose and reaching a significant decrease of about 20% with the highest dose vs. control cells. DRP1 content also showed a significant decrease (about −20%) in 500 µM palmitate-treated cells compared to lower doses (Figure 3B). Therefore, in cells treated with the highest dose, oleate induced a decrease in DRP1 content without changes in MFN2, suggesting a shift towards fusion, whereas palmitate induced a higher decrease (−30%) in Mfn2 than in DRP1 (−20%) content, suggesting a shift toward fission processes.

MFN2/DRP1 ratio, a marker of mitochondrial fusion/fission balance, showed a 19% increase in 500 μM oleate-treated cells compared to control cells. MFN2/DRP1 ratio was 1.07 ± 0.02 in 10 μM oleate-treated cells and 1.19 ± 0.02 in 500 μM treated cells. In contrast, palmitate induced a decrease (about −14%) in MFN2/DRP1 ratio in 500 μM treated cells, with a value of 0.86 ± 0.03 vs. control cells. Therefore, the highest dose elicited a 38% increase in MFN2/DRP1 ratio in oleate-treated cells compared to palmitate-treated cells.

**Figure 4:**
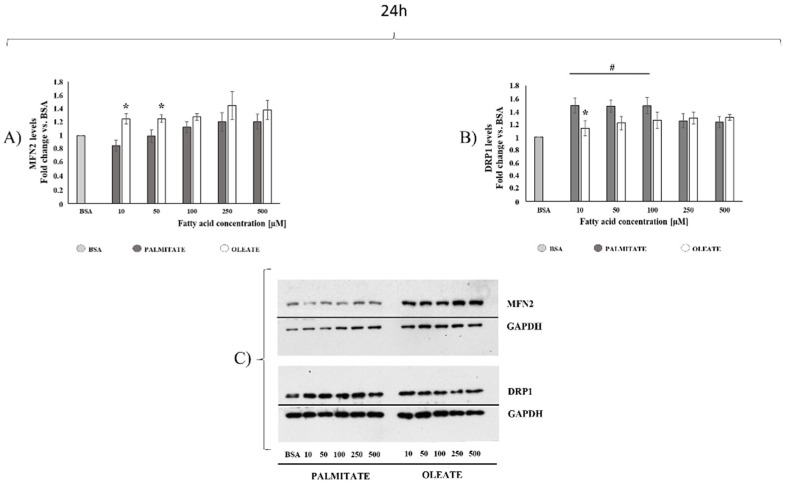
Mitochondrial dynamics markers after 24 h of exposure. Cells were treated with different doses of oleate or palmitate (0 μM, 10 μM, 50 μM, 100 μM, 250 μM and 500 μM) for 24 h. All data are presented as mean ± SEM for 5 biological replicates for MFN2 (**A**) and 6 biological replicates for DRP1 (**B**). (**A**) Regarding MFN2 content, two-way ANOVA analysis showed significant (*p* = 0.0139) concentrations effect, very significant (*p* = 0.0013) fatty acids effect, and no significant interaction. Student’s *t*-test: * *p* < 0.05 oleate vs. palmitate. (**B**) As for DRP1, two-way ANOVA analysis showed significant concentrations effect (*p* = 0.0041) and fatty acids effect (*p* = 0.0339), with no significant interaction. #*p* < 0.05 palmitate-treated cells vs. control cells according to a Tukey post hoc multiple comparison test. One-way ANOVA analysis confirmed a significant dose-dependent effect only for palmitate. Student’s *t*-test: * *p* < 0.05 oleate vs. palmitate. (**C**) Representative images of MFN2, DRP1 and loading control for protein normalization GAPDH were shown.

After 24 h, oleate induced a dose-dependent increase in MFN2 content, with increases of about +25% in 10, 50 and 100 μM treated cells, and further increases (about +40%) in 250 and 500 μM treated cells compared to control cells. On the other hand, palmitate induced a tendency to decrease (about −15%) in MFN2 content in the lowest dose (10 μM), but a not significant tendency to increase (about +20%) with high doses (250 μM and 500 μM) vs. control cells (Figure 4A). Student’s *t*-test analysis showed a significant MFN2 content increase (46% and 26%) in 10 μM and 50 μM oleate-treated vs. palmitate-treated cells, respectively (Figure 4A). The data herein suggested that low doses of oleate, but not palmitate, promoted an increase in mitochondrial fusion protein. We further analyzed the fusion process by testing OPA1 content, but no dose-dependent changes were found in palmitate- or oleate-treated cells after 24 h (Figure S1). As for fission processes, no significant changes in DRP1 content were observed with oleate, whereas palmitate induced a significant dose-dependent DRP1 increase. The increase was higher (about +48%) in cells treated with low doses (10 μM, 50 μM and 100 μM) of palmitate than in cells treated with high doses (about 24% increase with 250 μM and 500 μM palmitate) compared to control cells. Of note, 10 μM palmitate-treated cells showed a significantly higher (+31%) DRP1 content compared to 10 μM oleate-treated cells (Figure 4B). Therefore, oleate-treated cells showed a higher MFN2/DRP1 ratio than palmitate-treated cells, mainly with low doses. Indeed, MFN2/DRP1 ratio was 1.10 for oleate and 0.57 for palmitate in 10 μM treated cells, with an increase of 92% in oleate-treated cells, supporting the hypothesis of a shift towards fusion processes induced by oleate, but towards fission processes induced by palmitate with the lowest dose.

### 2.3. Comparison of Dose-Dependent Effects of Oleate and Palmitate on Caspase 3 Activity and GRP78 Contents after 24 h

**Figure 5:**
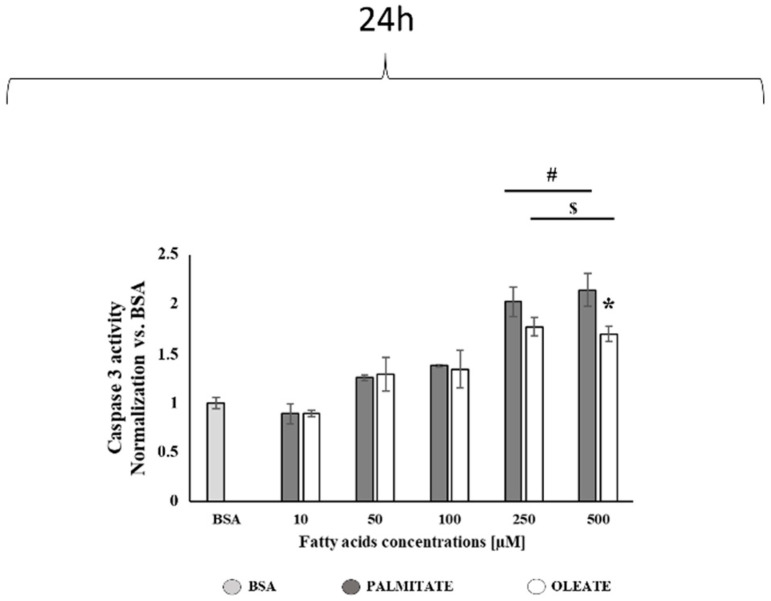
Dose-dependent effect of oleate or palmitate on caspase 3 activity after 24 h. Cells were treated with different doses of oleate or palmitate (0 μM, 10 μM, 50 μM, 100 μM, 250 μM and 500 μM) for 24 h. All data are presented as mean ± SEM for 3 or 4 biological replicates. Two-way ANOVA analysis showed that concentrations effect was extremely significant (*p* < 0.0001), fatty acids effect and interaction were not significant. # *p* < 0.05 palmitate vs. BSA and lower palmitate concentrations, $ *p* < 0.05 oleate vs. BSA and lower oleate concentrations according to a Tukey post hoc multiple comparison test. One-way ANOVA analysis confirmed the dose-dependent effect on caspase 3 activity for both palmitate and oleate with significant increases with the high doses. Student’s *t*-test: * *p* < 0.05 oleate vs. palmitate.

To test dose-dependent effects of oleate compared to palmitate on apoptosis after 24 h, analyses of caspase 3 activity, a protease which directly participates as effector of programmed cell death by apoptosis, were performed. Figure 5 showed that oleate elicited a dose-dependent effect on caspase 3 activity with a significant increase (about +70%) in 250 μM and 500 μM oleate-treated cells. Palmitate showed a similar trend but with a more marked significant increase of about twice the time in 250 μM and 500 μM palmitate-treated cells compared to control cells. Therefore, the caspase 3 activity increase was significantly lower (−30%) in 500 μM oleate-treated than in palmitate-treated cells.

**Figure 6:**
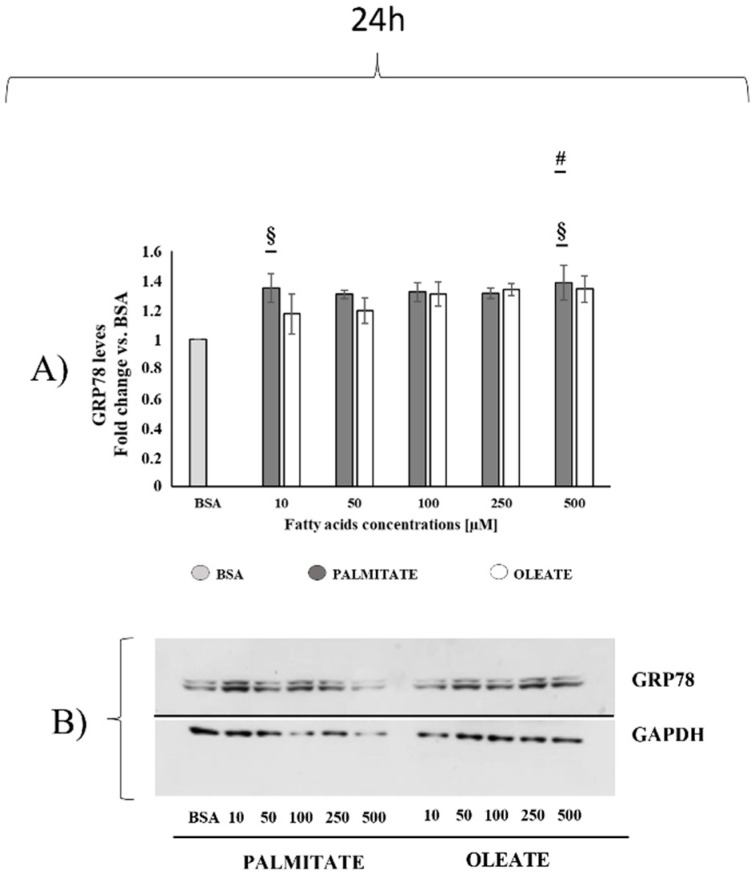
Dose-dependent effect of oleate or palmitate on glucose regulatory protein 78 (GRP78) content after 24 h. Cells were treated with different doses of oleate or palmitate (0 μM, 10 μM, 50 μM, 100 μM, 250 μM and 500 μM) for 24 h. (**A**) All data are presented as mean ± SEM for 4 biological replicates. Differences were evaluated for statistical significance by two-way ANOVA: concentration effect *p* = 0.0005 (extremely significant), fatty acids effect and interaction ns. # *p* < 0.05 palmitate vs. BSA according to a Tukey post hoc multiple comparison test. One-way ANOVA analysis showed that palmitate but not oleate induced significant dose-dependent changes in GRP78 content (§ *p* < 0.05 vs. BSA). (**B**) Representative images of GRP78 and loading control for protein normalization GAPDH.

After 24 h, we also tested the dose-dependent effects of oleate and palmitate on UPR by assessing the content of GRP78, a protein marker physiologically involved in ER protein folding regulation and UPR (Figure 6). Oleate did not induce any significant GRP78 increases in low-dose-treated cells vs. BSA, but a not significant tendency to increase (+35%) was observed in 250 and 500 μM treated cells vs. control. On the other hand, palmitate induced a dose-dependent increase in GRP78 content with a significant +35% increase in 10 and 500 μM treated cells vs. control (Figure 6). This finding suggested that UPR onset was induced by palmitate starting with the lowest dose.

## 3. Discussion

The aim of the present research work was to compare dose-dependent effects of oleate with palmitate on mitochondrial key proteins involved in fusion (MFN2) and fission (DRP1) processes by using two different treatment timings (8 and 24 h) in HepG2 cells. We also analyzed the effects on cell viability and lipid accumulation to compare oleate and palmitate lipotoxicity, and to evaluate whether diverse cytotoxic effects were associated with diverse mitochondrial dynamic behavior adaptive responses. To the best of our knowledge, the present study is the first to investigate the effect of monounsaturated fatty acids, namely oleate, on mitochondrial dynamics markers and cell viability in Hepg2 cells, in a dose- and time-dependent approach.

After 8 h of treatment, oleate did not induce any changes in cell viability, although it elicited a dose-dependent increase in cell lipid accumulation, more marked in 500 µM treated cells. Palmitate induced a similar increase in cell lipid accumulation, but it also elicited a significant decrease in cell viability in 500 µM treated cells. The maintenance of cell viability in 500 µM oleate-treated cells was associated with a decrease in DRP1 content without any change in MFN2 content with the consequent increase in MFN2/DRP1 ratio, a marker of mitochondrial fusion/fission balance, compared to both 500 µM palmitate-treated and control cells. This finding suggested an adaptive shift of mitochondrial dynamics towards fusion processes to support mitochondrial function under conditions of oleate oversupply. On the other hand, the decrease in both MFN2 content and MFN2/DRP1 ratio in 500 µM palmitate-treated cells suggested a shift towards fission processes induced by palmitate in association with a significant decrease in cell viability.

As expected, after 24 h of exposure, significant decreases in cell viability were found with lower fatty acids doses than after 8 h of exposure. A decrease in cell viability to the value of about 63% was induced by 500 µM palmitate after 8 h of exposure, and by 250 µM palmitate after 24 h. On the other hand, oleate did not have any effect after 8 h, whereas it induced a decrease in cell viability with the highest doses after 24 h. However, 250 and 500 µM oleate-treated cells showed a higher cell viability compared to palmitate-treated cells. Therefore, oleate treatments elicited lower cytotoxic effects compared to palmitate considering both fatty acid concentrations and time of exposure.

The higher cytotoxicity observed in palmitate-treated cells was associated with morphological alterations. A diverse lipid deposit mechanism could be hypothesized by morphological observations, although a similar increase in lipid content was found in both oleate- and palmitate-treated cells. Small lipid droplets were accumulated in the cytosol in association with morphological changes in palmitate-treated cells, which adopted a rounded shape, mainly after 24 h. On the contrary, large lipid droplets were observed in oleate-treated cells, without pronounced alterations in cell morphology. It should be noted that after 24 h, 500 µM oleate-treated cells showed a similar lipid accumulation, but a lower decrease in cell viability with no alterations in cell morphology compared to palmitate-treated cells. It has been shown that palmitic acid is preferably incorporated as diacylglycerols, whereas oleic acid is mainly accumulated as triacylglycerols, which are a neutral form of lipid storage. Moreover, it has been suggested that fatty acid incorporation in triacylglycerols could be protective against lipotoxicity and prevent impairments in mitochondrial function [36]. Therefore, the diverse mechanisms of lipid accumulation could explain the lower cytotoxic effect as well as the maintenance of a normal cell morphology in oleate-treated cells compared to palmitate-treated cells.

We further analyzed dose-dependent cell toxicity effects after 24 h by evaluating caspase 3 activity as a marker of apoptosis onset. Results showed that in line with the higher decrease in cell viability, a higher increase in caspase 3 activity was observed in 500 µM palmitate-treated cells compared to oleate-treated cells.

The differential effect of palmitate and oleate on cytotoxicity and apoptosis in a variety of cell types, including HepG2 cells, has been known for over a decade. However, given that conflicting data on oleic acid dose-dependent effects on cell viability were found in the literature, cell viability experiments were performed in the present experimental design to verify the cytotoxic effects of oleate under our experimental conditions. In agreement with our data, Chen et al. [35] showed that palmitic acid significantly reduced cell viability in a dose- and time-dependent manner in HepG2 cells treated with 200, 400 and 800 µM for 24 h or 48 h. In contrast to our data, oleic acid showed no toxicity at a concentration up to 0.8 mM after 24 h or 48 h exposure [35]. Moreover, after 24 h, concomitant incubation of palmitic and oleic acid at a mole ratio 2:1 (400 µM palmitic acid: 200 µM oleic acid) restored cell viability [35]. In neuronal cells, palmitic acid (400 µM) also induced dramatic cell apoptosis by increasing caspase 3 and caspase 8 activities, whereas 200 µM oleic acid did not induce increase in apoptosis makers, and its coincubation with palmitic acid blunted the apoptotic process [36]. Moreover, Kim et al. [37] observed a dose-dependent decrease in mesangial cells viability with palmitic acid doses ranging from 12.5 µM to 400 µM. They also showed an increase in cleaved caspase 3 content in cells treated with 100 µM of palmitic acid. Liu et al. [38] observed that palmitic acid induced a dose- and time-dependent deterioration in cell viability in a specific pancreatic cell line (INS-1E cells) with doses ranging from 100 to 800 µM, whereas oleic acid was not toxic. The combination of oleic and palmitic acid at a mole ratio of 1:1 (200 µM palmitic acid: 200 µM oleic acid) rescued the INS-1E cells from cell damage. These findings confirmed that saturated fatty acids are lipotoxic, whereas unsaturated fatty acids could be less toxic or even reduce apoptosis promoted by saturated fatty acids [35,37]. However, there are controversial data in the literature regarding oleic acid cytotoxicity in Hepg2 cells. In contrast to previously discussed data by Chen et al. [35], Zeng et al. showed no cytotoxic effect up to the 0.4 mM concentration, but observed a significant decrease in viability in cells treated with 0.8 mM of oleic acid [39]. In line with this finding, Mi et al. showed that HepG2 cell viability was not affected by up to 0.6 mM oleic acid in culture for 24 h, but 0.8 mM oleic acid significantly reduced cell viability [40]. Huang et al. showed significant cytotoxic effects with even high oleic acid doses (1 and 2 mM), with no effect up to 0.5 mM after 48 h [41]. In contrast with these findings, but in line with our present results, Pang et al. showed that after 24 h, cell viability significantly decreased in cells treated with 500 μM compared to control cells [42]. Moreover, Tian et al. showed significant decreases in cell viability in Hepg2 cells treated with 240, 480 and 960 μM for 24 h [43]. The different findings on oleic acid cytotoxic effects in the literature could be due to different experimental conditions, such as different types of culture medium or glucose/FBS concentrations in the culture medium. Our results were in line with the results of Pang et al., who used experimental conditions similar to those used in the present work, showing a significant decrease in cell viability associated with apoptosis increase in cells treated with 500 µM oleate for 24 h [42].

Given that apoptosis induction could be associated with ER homeostasis disruption and UPR onset, to further analyse the adaptive cell response to increasing doses of fatty acids after 24 h, we also evaluated endoplasmic reticulum stress onset by monitoring GRP78, as an UPR protein marker. This protein is associated with another three UPR sensors: activating transcription factor 6 (ATF6), inositol-requiring kinase 1 (IRE1) and protein kinase RNA-like endoplasmic reticulum kinase (PERK) and, when misfolded proteins are detected, GRP78 splits from those proteins and triggers the UPR [44,45]. In the present study, we focused only on the content of GRP78 as a marker of early ER stress and UPR onset, due to its role in cellular adaptive responses to restore ER and cellular homeostasis counteracting cellular death induction. Increases in GRP78 content could suggest an early ER stress onset. Our results showed that palmitate, rather than oleate, induced a significant dose-dependent increase in GRP78 protein content starting with the lowest dose (10 μM). With the limitation that other markers of ER stress should have been analyzed, it could be suggested that the increase in GRP78 without increase in caspase 3 activity could be an adaptive response useful to maintain cell homeostasis and viability in cells treated with a low palmitate dose. On the other hand, the increase in the UPR marker observed in 500 μM palmitate-treated cells associated with a marked increase in caspase 3 activity could suggest a further induction of ER stress leading to apoptosis, consistent with the significant reduction in cell viability. In agreement with this hypothesis, Chen et al. [35] showed that 400 μM palmitic acid induced a decrease in HepG2 cell viability associated with increased levels of ER stress markers, namely pPERK and ATF6. As for the oleate effect on GRP78 level, the present results showed that oleate did not induce a significant dose-dependent effect. In agreement with this finding, Chen et al. [35] demonstrated that 200 μM oleic acid did not induce increases in ER stress markers associated with no change in HepG2 cells viability. Moreover, they also showed that oleic acid supplementation alleviated palmitic-acid-induced ER stress in HepG2 cells [35].

As for the mitochondrial markers of fusion and fission processes after 24 h, diverse effects of oleate compared to palmitate on mitochondrial dynamic machinery were observed mainly in cells treated with low doses, whereas significant differences were observed only with the highest dose after 8 h. After 24 h, low palmitate doses induced a DRP1 content increase associated with a slight decrease in MFN2 content vs. control cells, with a consequent decrease in MFN2/DRP1 ratio. On the other hand, low oleate doses induced an increase in cellular MFN2 content with no significant changes in DRP1 content, suggesting a shift toward fusion processes. The increase in MFN2 content was also observed with high oleate doses. No changes were found in the content of OPA1 both in palmitate- and in oleate-treated cells. However, further experiments are needed to shed light on how OPA1 contributes to oleate and palmitate effects, by evaluating the content of different OPA1 isoforms that can play different roles.

The results obtained after 24 h of treatments allowed us to compare the effects of oleate and palmitate on several parameters associated with cell homeostasis, such as cell viability, lipid accumulation, mitochondrial dynamics, ER stress and apoptosis onset. Of note, with the lowest dose (10 μM) used in our study, no changes in cell viability and lipid accumulation were observed with both fatty acids, but a different cellular response was probably activated to maintain cellular homeostasis and viability. Indeed, the lowest dose of palmitate induced a shift in fusion/fission mitochondrial balance towards fission processes associated with induction of UPR, whereas the lowest dose of oleate induced a shift in the mitochondrial fusion/fission balance toward fusion process, with no UPR induction compared to palmitate. On the other hand, the highest doses of palmitate induced a similar increase in lipid accumulation but a higher decrease in cell viability associated with higher increase in caspase 3 activity and alteration in cell morphology compared to oleate-treated cells, where an increase in MFN2 content was observed starting by the low doses. Therefore, present results suggested that oleate, rather than palmitate, maintains mitochondrial dynamics without shifting towards fission processes and counteracting decreases in cell viability in Hepg2 cells. Indeed, taken together, our data on dose- and time-dependent experiments suggested that oleate promoted a shift toward mitochondrial fusion compared to palmitate, mainly with the highest dose after 8 h, but starting with low doses after 24 h of treatment. With the limitation that fusion and fission protein contents should also be assessed in isolated mitochondria and that microscopy observations are needed to confirm mitochondrial dynamics and morphology changes, present results suggested a different dose- and time-dependent effect of oleate from palmitate on mitochondrial dynamics protein markers in association with different effects on cell viability. Further studies performed by DRP1 silencing and/or MFN2 transfection on palmitate-mediated cell toxicity could also be useful to add further insight into the suggested mechanisms.

However, our results on palmitate induction of mitochondrial fission processes were in line with previous findings showing a shift towards fission processes in liver from rats fed a high lard diet rich in saturated fatty acids, where mitochondrial dynamics impairment was associated with mitochondrial dysfunction, hepatic steatosis and insulin resistance [16]. A decrease in Mfn2 and an increase in protein involved in fission processes (Drp1 and Fis1) accompanied by the presence of numerous small round mitochondria were observed in high lard diet fed rats vs. control rats [16]. A high lard diet also induced a shift toward mitochondrial fission in skeletal muscle [17] in line with previous reports that Mfn2 expression is reduced in skeletal muscle of obese Zucker rats and obese and diabetic patients [46,47]. Moreover, saturated fatty acids have also been reported to induce fission processes in vitro in differentiated C2C12 skeletal muscle cells [48] associated with mitochondrial dysfunction. Noteworthy, unsaturated fatty acids, namely omega 3 polyunsaturated fatty acids, have been shown to induce a shift towards a hepatic mitochondrial fusion phenotype associated with improvement of mitochondrial fatty acids utilization, which is useful to counteract hepatic lipid accumulation and insulin resistance in rats fed a high-fish-oil diet [16]. Moreover, omega 3 fatty acids reverted mitochondrial fission during in vitro hepatocyte steatosis through Mfn2 upregulation and tubular elongation [49].

To the best of our knowledge, the present results on oleate are the first to suggest a shift toward fusion processes induced by monounsaturated fatty acids. The mechanisms underlying the stimulation of mitochondrial fusion by unsaturated fatty acids, rather than saturated fatty acids, could involve changes in membrane lipid composition and fluidity among other factors. Further studies are needed to shed light on these mechanisms. However, in line with our hypothesis, Sparagna et al. showed images that resemble fusion in an oleate-treated cell and fission in a palmitate-treated cell in their research on the metabolic role for mitochondria in palmitate-induced cardiac myocyte apoptosis [50]. Moreover, it is interesting to note that Bourebaba et al. recently showed that HepG2 cell challenged 24 h with high doses of oleate/palmitate (2 mM, 2:1) exhibited lower levels of the fusion mediators and markedly higher levels of the mitochondrial fission mediators than normal healthy HepG2 cells [51]. They suggested that the observed mitochondrial dysfunction in lipotoxic HepG2 cells was related to both impaired fusion and enhanced fission. Moreover, the shift toward fission processes was associated with the upregulation of ER stress-related effectors, significant lipid accumulation and reduction in the average of the metabolically active living cells compared to control HepG2 cells [51]. These findings underlined the importance of dose-dependent studies to shed light on adaptive cell response to stressors agents, such as dietary fatty acids.

Our data on the different adaptive response in terms of mitochondrial fusion/fission balance in oleate-treated compared to palmitate-treated cells could suggest that: (1) the dose- and time-dependent adaptive cellular response relies on which type of dietary fatty acid has been used; (2) oleate protective effects towards cell stress and toxicity could involve mitochondrial dynamics behavior closely related to mitochondrial metabolic function. This finding could be useful to understand the role of mitochondria in the protective effects of bioactive compounds, such as oleate. Further experiments are needed to clarify the link among fatty acid dose- and time-dependent changes in fusion/fission balance, hepatic steatosis and insulin resistance onset in HepG2 cells.

## 4. Materials and Methods

### 4.1. Cell Culture

Human hepatocellular carcinoma cells (HepG-2) were obtained from Interlab Cell Line Collection (Centro di Biotecnologie Avanzate, Genova, Italy). HepG-2 cells were cultured in 100 × 10 mm Petri dishes in Minimum Essential Medium (MEM) supplemented with 10% fetal bovine serum (*v/v*), 1% (*v/v*) non-essential amino acids, 0.2 mM L-glutamine, 50 units/mL penicillin and 50 μg/mL streptomycin (Invitrogen SRL, Milan, Italy). Cells were maintained at 37 °C in a 5% CO_2_, 95% air-humidified atmosphere and passed twice a week. Cells were then treated with saturated (sodium palmitate) or monounsaturated (sodium oleate) fatty acids (Merck, Darmstadt, Germany) conjugated with bovine serum albumin (BSA). Briefly, cells were plated at 5 × 104 cell/cm^2^ and then cultured for 8, 24 or 48 h in the medium containing palmitate or oleate at five different doses: 10 μM, 50 μM, 100 μM, 250 μM and 500 μM. This dose range was chosen based on values reported for in vitro studies on mammalian cell models [33,35,52]. In all cases, cells did not exceed 70% confluence at the time of the treatment.

### 4.2. Preparation of BSA-Conjugated Free Fatty Acids Solutions

Palmitate and oleate 100 mM stock solutions were prepared in NaOH 0.1 M and were dissolved at 70 °C for 15 min under stirring. A 10% BSA fatty acid free (Merck, Darmstadt, Germany) solution was dissolved at 55 °C in NaCl 0.9 % and mixed into the 100 mM fatty acids solutions and maintained for more than 15 min in a shaking water bath at 55 °C. Then, the stock solutions were stored at −20 °C and used, at the time of treatment, to prepare the final concentration of chosen experimental doses (500 µM, 250 µM, 100 µM, 50 µM and 10 µM) of palmitate and oleate.

### 4.3. Cell Viability

Cell viability was determined by using 3-(4.5-dimethyl-2-thiazolyl)-2.5-diphenyl-2H-tetrazolium bromide (MTT) method (M5655, Merck, Darmstadt, Germany), which is a colorimetric assay based on the enzymatic conversion of MTT (yellow) into formazan (purple) by metabolically active cells, indicating the quantity of living cells [52]. After incubation with palmitate or oleate, 0.5 mg/mL of MTT was added to 100 µL of cell medium in a 96-well plate and incubated for 1 h 30 min at 37 °C and 5% CO_2_ to allow MTT to be metabolized. The resulting formazan crystals were dissolved in dimethyl sulfoxide (DMSO) and absorbances were measured in a microplate reader at 595 and 655 nm, providing the number of living cells.

### 4.4. Oil Red Stain

Oil red stain quantitative (spectrophotometric) and qualitative (microscopic) analyses were performed using a classic protocol to detect lipid droplets in cells [53]. Briefly, after treatments, cells were washed in PBS at 37 °C, fixed in 4% PFA for 45min at room temperature, washed twice with distilled water and stained as indicated by Oil-Red-O conventional protocols. To quantify Oil-Red-O accumulated in cells, the stain was extracted from cell lipid droplets using isopropanol 100% and measured by using a 96-well plate reader at 490 nm. Quantitative and qualitative analyses were compared to non-stimulated cells (BSA).

### 4.5. Western Blot Analysis

As mentioned above, cells were cultured for 24 h in medium containing palmitate or oleate conjugated with BSA at five different doses: 10 μM, 50 μM, 100 μM, 250 μM and 500 μM. At the end of the treatment, medium was discarded and cells were washed twice with ice-cold phosphate buffered saline (PBS), and then were mechanically harvested and lysed with lysis buffer containing 50 mM Tris-HCl pH 7.5, 150 mM NaCl, 1 mM EDTA, 0.1% sodium dodecyl sulphate (SDS), 1% Triton X-100, 1 mM ortovanadate, 2 mM PMSF, 10 mM NaF and the inhibitor cocktail consisting of 104 lM 4-(2-aminoethyl) benzenesulfonyl fluoridehydrochloride, 80 nM aprotinin, 4 lM bestatin, 1.4 lM E-64.2.0 lM leupeptin and 1.5 lM pepstatin A (Merck, Darmstadt, Germany).

After 30 min of incubation on ice, cell extracts were centrifuged at 13,000× *g* for 20 min at 4 °C, to remove cell debris. The protein concentration on lysates was measured with Bio-Rad Protein Assay Reagent (Bio-Rad, Hercules, CA, USA) using a BSA curve as a standard and about 30 µg of total proteins was separated by using sodium dodecyl sulfate–polyacrylamide gel electrophoresis. At the end of separation, protein bands were transferred to a PVDF blotting membrane (GE Healthcare, Buckinghamshire, UK). The blots were then incubated with 5% skim milk in Tris-buffered saline (TBS) for 60 min, and then further incubated overnight at 4 °C with the primary antibodies of interest: Mitofusin 2 (Mfn2, sc-100560,1:1000; Santa Cruz Biotechnology, Heidelberg, Germany), DRP1 (sc-3298, 1:1000; Santa Cruz Biotechnology, Heidelberg, Germany), GRP78 (sc-1050, 1:1000, Santa Cruz Biotechnology, Heidelberg, Germany) or OPA-1 (sc-30572, 1: 1000, Santa Cruz Biotechnology, Heidelberg, Germany).

The second day, each membrane was washed three times with TBS-Tween (15 min for each one) and incubated for 1 h at room temperature with the specific secondary antibody: anti-mouse, anti-rabbit (1:10,000; Bio-Rad, Hercules, CA, USA); or anti goat peroxidase secondary antibody (1:1000, Santa Cruz Biotechnology, Heidelberg, Germany).

Immunocomplexes were revealed using a chemiluminescence detection kit (Immobilon Western, Millipore, Dublin, Ireland) according to the manufacturer’s instructions. To normalize the amount of each protein in the total cellular extract, anti- glyceraldehyde 3-phosphate dehydrogenase (GAPDH) (ab8245, 1:2000, Abcam, Cambridge, UK) was used as loading control guide. Quantification of immunoblot films was performed with Quantity One 1-D Analysis Software (Bio-Rad, Hercules, CA, USA).

### 4.6. Caspase-3 Assay

To evaluate the apoptosis process, enzymatic Caspase-3 activity assay was performed according to the manufacturer instructions (Caspase 3 Assay Kit, Colorimetric; CASP-3-C, Merck, Darmstadt, Germany).

### 4.7. Statistics

Statistical analysis was performed using GraphPad prism 8.3.1 (GraphPad software Inc. San Diego, CA, USA). Data are reported as mean ± standard error of the mean (SEM). Two-way ANOVA analysis followed by Tukey’s post hoc test was used to evaluate the effect of the different fatty acids (palmitate or oleate) used to treat cells and the effect of different concentrations (0, 10, 50, 100, 250, 500 µM) tested in this study. One-way ANOVA analysis and Student’s *t*-test was used to further analyze fatty acids effects. *p* values ≤ 0.05 were considered to be statistically significant. In Western blot analysis, figures show representative blots.

## Data Availability

Our own data presented in this study are available on request from the corresponding author.

## Supplementary Materials

The following are available online at https://www.mdpi.com/article/10.3390/ijms22189812/s1.
