# Supplementary material for: Dose- and Time-Dependent Effects of Oleate on Mitochondrial Fusion/Fission Proteins and Cell Viability in HepG2 Cells: Comparison with Palmitate Effects
Source: Int J Mol Sci. 2021 Sep 10;22(18):9812. doi: 10.3390/ijms22189812 (PMC8468319; doi:10.3390/ijms22189812)
Supplement: Supplementary file 1 [file ijms-22-09812-s001.zip › ijms-1359573-supplementary.pdf]

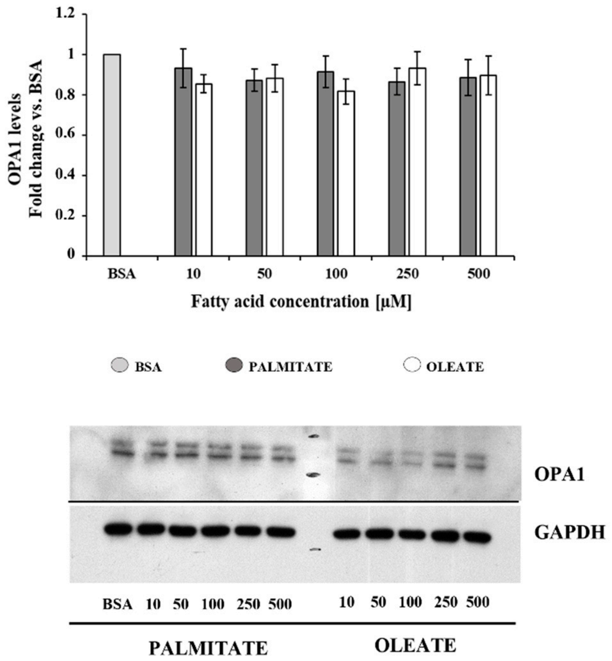

Figure S1. Dose dependent effect of oleate or palmitate on optic atrophy 1 (OPA1) protein content after 24 hours. Cells were treated with different doses of oleate or palmitate (0 µM, 10 µM, 50 µM, 100 µM, 250 µM and 500 µM) for 24 hours. A. All data are presented as mean ± standard error of the mean (SEM) for 4 biological replicates. Differences were evaluated for statistical significance by two-way ANOVA: concentration effect ns., fatty acids effect ns. and interaction ns. One-way ANOVA analysis showed no significant difference. B. Representative images of OPA1 and loading control for protein normalization glyceraldehyde 3-phosphate dehydrogenase (GAPDH).
